# TRP Channels as Drug Targets to Relieve Itch

**DOI:** 10.3390/ph11040100

**Published:** 2018-10-06

**Authors:** Zili Xie, Hongzhen Hu

**Affiliations:** Department of Anesthesiology, The Center for the Study of Itch, Washington University School of Medicine, St. Louis, MO 63110, USA; zilixie@wustl.edu

**Keywords:** TRP channels, itch, pain, TRPA1, TRPV1, TRPV3, TRPV4, TRPM8, TRPC4, agonists, antagonists

## Abstract

Although acute itch has a protective role by removing irritants to avoid further damage, chronic itch is debilitating, significantly impacting quality of life. Over the past two decades, a considerable amount of stimulating research has been carried out to delineate mechanisms of itch at the molecular, cellular, and circuit levels. There is growing evidence that transient receptor potential (TRP) channels play important roles in itch signaling. The purpose of this review is to summarize our current knowledge about the role of TRP channels in the generation of itch under both physiological and pathological conditions, thereby identifying them as potential drug targets for effective anti-itch therapies.

## 1. Introduction

Itch, also known as pruritus, is an unpleasant sensation provoking the scratch reflex [1]. Itch is classified as acute or chronic, with the latter defined as pruritus lasting longer than six weeks [2]. Acute itch is often generated locally in the skin by pruritogens and the scratching behavior that is evoked has a protective role by removing irritants. Chronic itch, by contrast, may be maladaptive and leads to significant decrements in quality of life. It is associated with numerous conditions, including atopic eczema, psoriasis, urticaria, inflammatory skin diseases, chronic renal failure, cholestasis, lymphoma, and chronic liver diseases [3,4,5,6]. Chronic itch remains an unmet medical condition lacking universally effective treatments. Delineating itch mechanisms at the molecular, cellular, and circuit levels is critical to the development of new anti-itch therapeutic strategies.

The transient receptor potential (TRP) channels comprise 28 members in mammals that can be divided into six subfamilies based on amino acid sequence homology, including TRPA, TRPC, TRPM, TRPML, TRPP, and TRPV [7]. TRP channels respond to a diverse array of thermal, chemical, and mechanical stimuli. They are implicated in many sensory functions including taste, smell, thermoception, touch, osmolarity, and pain [8,9,10,11,12]. In the past two decades, numerous studies have demonstrated that TRP channels are critically involved in itch sensation under both physiological and pathological conditions [13,14].

## 2. Cellular Mechanisms of Itch Signaling in the Periphery

Itch has long been considered a submodality of pain based on observations that local stimulation of the same skin sites at low intensities evokes itch, whereas high intensities cause pain [15]. Pain and itch are now clearly understood to be distinct sensory modalities [16,17,18]. Pruritogens evoke itch sensation by activating pruriceptors present on free nerve endings of cutaneous primary sensory neurons [19]. Primary sensory neurons are classified into four distinct types based on cell body size and axon myelination: (1) large-diameter, thickly myelinated proprioceptive neurons; (2) large-diameter, myelinated Aβ low-threshold mechanoreceptors which mediate touch; (3) medium-sized, lightly myelinated Aδ nociceptive neurons; and (4) small-diameter, unmyelinated C nociceptive neurons that detect noxious stimuli [19]. LaMotte et al. showed that itch-initiating neurons are predominantly C-type neurons with an additional small population of Aδ neurons [20]. Thus, both pain and itch are transduced by small-diameter dorsal root ganglion (DRG) neurons. However, the two modalities are transduced separately by pain-selective (nociceptive) and itch-selective (pruriceptive) neuronal subpopulations in the skin. This is firmly supported by the finding that the ablation of Mas-related G protein-coupled receptor member A3 (MrgprA3) neurons substantially reduces scratching evoked by multiple pruritogens and under chronic itch conditions, leaving pain sensitivity intact [21].

**Figure 1:**
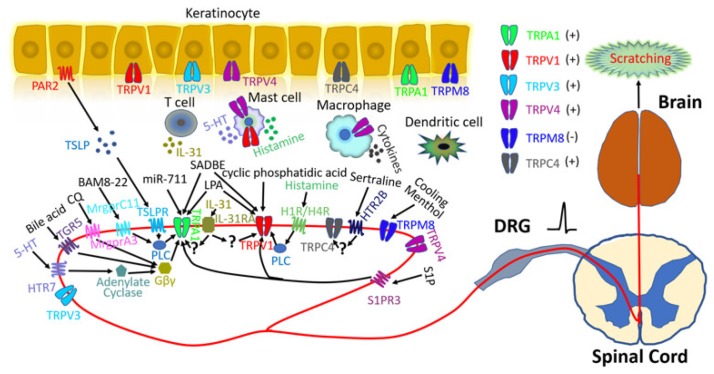
Transient receptor potential (TRP) channels and itch signaling. Six TRP channels are implicated in itch signaling, including TRPA1, TRPV1, TRPV3, TRPV4, TRPM8, and TRPC4. All itch-related TRP channels are expressed in keratinocytes and/or primary sensory neurons. TRPA1 is required for itch evoked by chloroquine (CQ), bovine adrenal medulla 8–22 peptide (BAM8-22), 5-hydroxytryptamine (5-HT), bile acid, and thymic stromal lymphopoietin (TSLP) which can be released from keratinocytes through protease-activated receptor 2 (PAR2) activation. These pruritogens bind to their respective G protein-coupled receptors (GPCRs): Mas-related G protein-coupled receptor member A3 (MrgprA3), MrgprC11, 5-hydroxytryptamine receptor 7(HTR7), G protein-coupled bile acid receptor Gpbar1 (TGR5), and thymic stromal lymphopoietin receptor (TSLPR). Phospholipase C (PLC) and Gβγ signaling downstream from these receptors contribute to TRPA1 activation. TRPA1 also mediates miR-711-induced itch through direct activation. TRPV1 is required for histamine-evoked itch, whereby histamine receptor 1 (H1R) and histamine receptor 4 (H4R) activate TRPV1 through the PLC signaling pathway. TRPV1 also participates in cyclic phosphatidic acid-induced itch through direct activation. Both TRPA1 and TRPV1 are involved in squaric acid dibutylester (SADBE)- and lysophosphatidic acid (LPA)-induced itch through direct activation. In addition, both TRPA1 and TRPV1 are required for interleukin-31 (IL-31)- and sphingosine 1-phosphate (S1P)-related itch, but the detailed mechanisms remain unresolved. Mutations of amino acid residues in TRPV3 have been associated with Olmsted syndrome (OS), suggesting a potential role in itch signaling. Emerging data implicate TRPV4 in both histaminergic and non-histaminergic itch, but results from different research groups are controversial [27,28,29]. HTR2B and TRPC4 are involved in selective serotonin reuptake inhibitor (SSRI)-evoked pruritus. Unlike other TRP channels, TRPM8 activation inhibits itch and is required for cooling- and menthol-mediated itch inhibition.

In addition to sensory neurons, many other cell types are also involved in itch pathways [22]. For instance, stimulated keratinocytes release numerous inflammatory mediators including thymic stromal lymphopoietin (TSLP), ATP, endothelins, prostaglandins, histamine, nitric oxide, and serotonin which can directly activate or sensitize primary sensory neurons to initiate itch [22]. Similarly, innate immune cells (such as mast cells, neutrophils, macrophages, and dendritic cells) release a versatile ensemble of pruritogenic inflammatory mediators [23]. The interplay between adaptive immune cells and neurons also plays an important role in itch sensation (Figure 1) [24,25,26].

## 3. TRP Channels and Itch Signaling

TRP channels are molecular sensors of mechanical, chemical, and thermal environmental cues and are crucially involved in both acute and chronic itch [19,22,30,31,32,33,34]. Six TRP channels are now firmly associated with itch generation and transduction (Figure 1).

### 3.1. TRPA1-Dependent Itch

TRPA1 is named after the 14 ankyrin repeats in its cytosolic N-termini; it is the sole member of the TRPA subfamily in mammals [35]. TRPA1 is selectively expressed by a subpopulation of small-diameter neurons in dorsal root, trigeminal, and nodose ganglia and keratinocytes [36,37,38]. TRPA1 is directly activated by a vast array of noxious and electrophilic compounds (such as allyl isothiocyanate, cinnamaldehyde, allicin, and diallyl disulfide) [39,40,41], and numerous endogenous reactive oxygen species (such as hydrogen peroxide and 4-hydroxynonenal (4-HNE) [42,43]. It can also be activated by the transition metal ion Zn^2+^, leading to acute pain [44].

Itch-related G protein-coupled receptors (GPCRs), including the bile acid receptor TGR5, the TSLP receptor, and the MrgprA3 and MrgprC11, can positively modulate TRPA1 [45,46,47]. Many pharmacological blockade and genetic ablation studies have shown that TRPA1 is a critical downstream mediator of GPCR signaling in different mouse models of acute and chronic itch.

#### 3.1.1. TRPA1 in Histamine-Independent Itch Induced by Chloroquine (CQ) and Bovine Adrenal Medulla 8–22 Peptide (BAM8-22)

Antihistamines are useful for treating many types of acute itch, but generally ineffective under many chronic itch conditions, which are histamine-independent. CQ is commonly used to treat malaria and evokes severe itching as a major side effect, which is especially common among black Africans. Liu et al. found that CQ activated MrgprA3 to trigger histamine-independent itch [46]. Both loss- and gain-of-function studies further demonstrated that MrgprA3 was required for CQ-induced itch in mice [46]. Similarly, MrgprC11 was required for BAM8-22-induced acute itch in mice [46]. Moreover, Wilson et al. showed that TRPA1 was a downstream target of both MrgprA3 and MrgprC11 signaling and required for both CQ- and BAM8-22-induced itch [48]. However, distinct mechanisms are involved in MrgprA3 and MrgprC11 signaling to activate TRPA1: Gβγ signaling is required for MrgprA3 and phospholipase C (PLC) signaling is required for MrgprC11 (Figure 1) [48]. Interestingly, a recent study demonstrated that CQ-evoked primary afferent firing ex vivo, and scratching responses in vivo, were not significantly different between wild-type and TRPA1-deficient mice. Instead, chloride channels may play a more important role in CQ-induced increases in sensory neuron excitability and scratching [49].

#### 3.1.2. TRPA1 in 5-Hydroxytryptamine (5-HT)-Induced Itch

5-HT is released by tissue-resident mast cells, which can in turn activate pruriceptive afferents [50]. Pruritogenic 5-HT signaling occurs in some localized or systemic diseases, such as atopic dermatitis (AD), cholestasis, and psoriasis [51]. Morita et al. found that both HTR7 and TRPA1 were required for 5-HT-induced itch because mice lacking HTR7 or TRPA1 had severely reduced scratching responses in both 5-HT-induced acute itch and in a mouse model of AD (Figure 1) [52]. On the other hand, Akiyama et al. showed that 5-HT-induced acute scratching required TRPV4 but not TRPA1 or TRPV1 because scratching was significantly diminished in TRPV4 knockout (KO) mice, but unaffected by genetic ablation of either TRPA1 or TRPV1 function [27].

#### 3.1.3. TRPA1 in Cholestatic Pruritus

Patients with cholestatic or other obstructive liver diseases often suffer severe pruritus caused by increased levels of bile acid in the circulation and skin [53]. Indeed, bile acid chelators can relieve cholestatic pruritus, whereas direct intradermal injections of bile acid cause scratching in mice [54,55]. Lieu et al. demonstrated that TGR5 activates and sensitizes TRPA1 by a Gβγ- and PKC-dependent mechanism and that bile acid-induced itch requires TRPA1 (Figure 1) [45]. Both pharmacological inhibition and genetic ablation of TRPA1 function prevented the bile acid-stimulated release of the pruritogenic neuropeptide gastrin-releasing peptide (GRP) and reduced the bile acid-elicited scratching. Moreover, spontaneous scratching was exacerbated in transgenic mice overexpressing TGR5, which was prevented by the administration of a TRPA1 antagonist [45]. Thus, TRPA1 and TGR5 are potential drug targets for alleviating itch in patients with cholestatic and other obstructive liver diseases.

#### 3.1.4. TRPA1 in Itch Associated with AD

The protease-activated receptor 2 (PAR2) plays an important role in keratinocyte TSLP production, and there is a correlation between PAR2 activity and TSLP expression in the skin of AD patients and in mouse models of atopic diseases [56]. Interestingly, TSLP over-expression in keratinocytes triggers robust itch-evoked scratching and the development of an AD-like skin phenotype in mice [57]. Wilson et al. reported that PAR2 evoked robustly increased expression of TSLP in keratinocytes and that TSLP acted directly on a TRPA1-positive subset of sensory neurons to produce robust scratching via TSLP receptor (TSLPR) signaling [47]. Subcutaneous TSLP injections in wild-type mice evoked scratching which was significantly attenuated by functional ablation of TRPA1 and by genetic ablation of IL7Rα which formed a heterodimeric receptor with TSLPR, but not by functional ablation of TRPV1. PLC signaling was required for the functional coupling between TSLPR signaling and TRPA1 activation (Figure 1) [47].

#### 3.1.5. TRPA1 in Itch Induced by Extracellular miRNAs

Chronic itch is a major symptom of cutaneous T cell lymphoma [58]. Intradermal inoculation of human Myla cells can induce lymphoma associated with intense itching in immune-deficient mice [59]. Interestingly, lymphoma-associated itch is suppressed by a miRNA-711 inhibitor and a blocking peptide that disrupts miRNA-711/TRPA1 interaction. Moreover, extracellular miRNA directly activates TRPA1 on sensory neurons to induce TRPA1-depedent itch without causing pain in naive mice (Figure 1) [59]. These findings demonstrated an unconventional physiological role of extracellular naked miRNAs as pruritogens and TRPA1 modulators [59,60].

### 3.2. TRPV1-Dependent Itch

TRPV1 belongs to a subfamily of temperature-sensitive TRP channels, also called “ThermoTRPs” [61]. TRPV1 is activated by noxious temperatures (>43 °C) [62]. In addition, TRPV1 is activated by capsaicin, low pH, and numerous molecules associated with inflammation and tissue damage such as bradykinin, prokineticin, prostaglandins, anandamide, and retinoids [22,63,64,65].

Histamine is one of the best-known endogenous pruritogens [66]. Applied to human skin, histamine causes local vasodilation and gives rise to the characteristic triple response: redness, flare, and swelling, accompanied by intense itching [67]. Among four histamine receptors (including the histamine receptors H1R, H2R, H3R, and H4R), H1R and H4R are expressed by pruriceptive DRG neurons and mediate histamine-induced itch [68]. TRPV1 plays a crucial role in histamine-induced itch via coupling with H1R-mediated signaling [69]. Histamine activates inward currents only when H1R and TRPV1 are co-expressed heterologously, and histamine evokes Ca^2+^ influxes in sensory neurons isolated from wild-type but not TRPV1 KO mice [69]. Importantly, TRPV1 KO mice have markedly reduced histamine-induced scratching compared to wild-type mice [69]. In addition, TRPV1 may be involved in histamine-induced itch mediated by H4R (Figure 1) [70].

Besides histamine-dependent itch, direct activation of TRPV1 by the pruritogen, cyclic phosphatidic acid, also evokes robust scratching responses [71]. The naturally occurring monounsaturated fatty acid and oleic acid antagonize TRPV1 by interacting with the vanilloid (capsaicin)-binding pocket and promoting stabilization of a closed state conformation. Importantly, oleic acid inhibits cyclic phosphatidic acid-induced activation of TRPV1 in vitro as well as TRPV1-dependent pain and itch responses in vivo [71], further highlighting the importance of TRPV1 in itch sensation (Figure 1).

### 3.3. Itch Mediated by Both TRPA1 and TRPV1

Lysophosphatidic acid (LPA) is another proposed itch mediator besides bile acid in cholestatic pruritus [72]. Intradermal injections of LPA provoke scratching behaviors in rodents [72,73,74]. Both TRPA1 and TRPV1 are implicated in LPA-induced itch (Figure 1). Genetic ablation of either TRPV1 or TRPA1 reduces LPA-induced activation of DRG neurons and scratching responses elicited by intradermal LPA injections [74]. LPA directly activates TRPA1 through interactions with positive charged amino acids KK672–673/KR977–978 and K710 [74,75] on the cytosolic sites of the protein.

Squaric acid dibutylester (SADBE) is a small molecule hapten commonly used for treating alopecia areata and warts that often causes contact hypersensitivity (CHS) in humans [76,77]. SADBE effectively induces CHS in a mouse model of allergic contact dermatitis (ACD) associated with spontaneous scratching behaviors [78]. Recently, Feng et al. found that both TRPA1 and TRPV1 were involved in generating spontaneous scratching in SADBE-induced ACD model in mice (Figure 1) [30]. Unexpectedly, TRPV1 but not TRPA1 protected against the SADBE-induced skin inflammation, suggesting that chronic skin inflammation and persistent itch in SADBE-induced CHS were mediated by distinct molecular mechanisms. These results suggested that extreme care should be taken when applying TRP channel blockers to treat chronic itch because they might also promote skin inflammation.

T-cell-derived Th2 cytokine IL31 is upregulated in itch-related cutaneous T cell lymphoma and AD in humans [4,79]. The neutralization of interleukin-31 (IL-31) by anti-interleukin-31 antibodies ameliorates scratching behavior in the NC/Nga mouse model of AD (a strain of Japanese fancy mice) [80]. Moreover, cutaneous or intrathecal injections of IL-31 provoke intense itching, and IL-31 concentration is increased significantly in AD-like skin preparations in mice [81]. Both human and mouse DRG neurons express IL-31RA, primarily in neurons that co-express TRPV1. Compared with respective control mice, IL-31-induced itch response is significantly attenuated in either TRPV1 or TRPA1 KO mice [81]. Taken together, it is likely both TRPA1 and TRPV1 are involved in IL-31-induced itch (Figure 1). Thus, both TRPA1 and TRPV1 represent possible therapeutic targets for the management of Th2 cytokine-mediated itch.

Sphingosine 1-phosphate (S1P) is an important signaling sphingolipid involved in both itch and pain signaling [82,83,84,85]. A recent study showed that low concentrations of S1P selectively evoked acute itch, and that high concentrations of S1P caused both pain and itch [82]. S1P receptor 3 (S1PR3) appeared to be the sole mediator of both S1P-induced itch and pain responses through promoting excitability of different primary afferents: S1PR3 and TRPA1 co-existed in a subset of pruriceptors, but S1PR3 was co-expressed with TRPV1 in a subset of heat nociceptors. In line with these unique expression patterns, TRPA1 mediated S1P-induced itch behaviors and TRPV1 mediated S1P-elicited acute pain and heat hypersensitivity (Figure 1). These findings suggested that distinct cellular and molecular mechanisms mediated S1P-induced itch and pain sensations [82].

### 3.4. TRPV3-Dependent Itch

TRPV3 is another ThermoTRP that is activated by temperatures >33 °C [86]. It is activated by many endogenous ligands (such as farnesyl pyrophosphate) [87], synthetic compounds (such as 2-aminoethoxydiphenyl borate (2-APB)) [88], and some natural compounds (such as carvacrol, menthol, thymol, eugenol, and citral) [89,90,91,92].

Unlike TRPA1 and TRPV1 channels, which are mainly expressed by primary sensory neurons, TRPV3 is primarily expressed by mouse skin keratinocytes and oral and nasal epithelia, although TRPV3 is also detected in human DRG neurons (Figure 1) [93,94,95]. TRPV3 expression is increased in keratinocytes from AD lesions and patients suffering from postburn pruritus [96,97], but not in biopsies from chronic idiopathic pruritus (CIP) patients [98].

TRPV3 is widely involved in skin barrier function, hair growth, skin inflammation, pain, and itch [13,99,100,101]. The role of TRPV3 in itch sensation attracts a great deal of attention since Asakawa et al. first reported that the Gly573Ser substitution in Trpv3 gene (TRPV3*^Gly573Ser^*) in DS-Nh mice produced a hairlessness phenotype and that these mice also developed allergic and pruritic dermatitis [102]. Both G573S and G573C mutations of murine TRPV3 are gain-of-function mutations promoting basal TRPV3 channel activity and enhancing responses to TRPV3 ligands compared to wild-type TRPV3 [103]. These mutations drive constitutive TRPV3 activity under normal physiological conditions, subsequently altering ion homeostasis and membrane potentials of skin keratinocytes, leading to hair loss and dermatitis-like skin phenotypes.

Gain-of-function mutations in human TRPV3 from patients with Olmsted syndrome (OS) suggest a role of TRPV3 in human itch signaling [104]. OS is a rare skin disease characterized by palmoplantar, alopecia, onychodystrophy, and severe itching [105]. To date, mutations of five different amino acid residues in TRPV3 have been associated with OS from more than 70 cases, including p.Gly568, p.Gly573, p.Leu673, p.Trp692, and p.Asn415_Arg416 [106].

Although TRPV3 mutants in both DS-Nh mice and OS patients display itch symptoms, it remains unclear whether itch arises directly from TRPV3-mediated signaling or secondarily through TRPV3-mediated skin pathogenesis. Direct evidence supporting the involvement of TRPV3 in itch signaling is currently lacking. It is possible that additional cellular and molecular mechanisms are required to produce itch in TRPV3 mutants in vivo, besides elevated basal activity and altered ligand-evoked responses.

### 3.5. TRPV4-Dependent Itch

TRPV4 is another ThermoTRP activated at temperatures between 27 °C and 35 °C [107]. TRPV4 expression occurs in skin, kidney, urinary bladder, and DRG [108,109]. Interestingly, photodermatitis and sunburn are accompanied by symptoms of pain and enhanced epidermal expression of TRPV4 [110], suggesting TRPV4 may be a sunburn pain mediator.

Recently, Akiyama et al. reported that TRPV4 played an important role in 5-HT-induced acute itch because 5-HT- but not the peptide Ser-Leu-Ile-Gly-Arg-Leu (SLIGRL)- or histamine-evoked scratching was reduced in TRPV4 KO mice, compared with wild-type mice [27]. However, Chen et al. showed that scratching responses elicited by all histaminergic pruritogens including histamine, compound 48/80, and ET-1 were significantly attenuated in both global and keratinocyte-specific TRPV4 KO mice compared with their respective controls [28]. In line with these findings, Kim et al. showed that TRPV4 was required for histamine-induced itch because TRPV4 KO mice exhibited significant reductions in scratching responses to histamine compared with wild-type littermates [29].

The role of TRPV4 in scratching responses induced by non-histaminergic pruritogen CQ is controversial. Akiyama et al. showed that the number of CQ-induced scratching bouts was significantly increased in global TRPV4 KO mice compared with wild-type mice [27]. In contrast, Chen et al. found that CQ-evoked scratching was unaffected by global TRPV4 KO or skin-specific TRPV4 KO, compared with their respective controls [28]. Even more surprisingly, Kim et al. showed that global TRPV4 KO mice exhibited a significant attenuation in scratching responses to CQ compared with wild-type littermates [29].

Besides involvement in acute itch, Luo et al. reported that lineage-specific deletion of TRPV4 in macrophages and keratinocytes reduced both allergic and nonallergic chronic itch in mice [98]. Additionally, TRPV4 expression in skin biopsies from CIP patients was significantly increased compared with healthy controls. Taken together, the evidence implicates TRPV4 in both allergic and non-allergic chronic itch, and thus represents a potential therapeutic target for both conditions.

### 3.6. TRPM8 in Itch Modulation

TRPM8 is activated by cool and cold temperatures between 8 °C and 28 °C [111,112]. It can also be activated by several natural compounds that can lead to a cooling sensation, including menthone, menthol, and eucalyptol [113,114]. TRPM8 is expressed by keratinocytes and a subset of primary afferent sensory neurons and serves as a cold sensor in vivo [115,116,117].

Pain, itch, and cold sensations are closely related. Under certain circumstances, pain can inhibit itch and cooling can relieve both pain and itch [118,119,120,121,122]. Cooling by applying ice or cold water is a commonly used method to relieve itch [121]. Emerging evidence suggests that TRPM8 activation reduces itch, whereas some specific TRPM8 activators can aggravate itch [123,124]. Icilin, a potent TRPM8 activator, is effective in reducing vulva pruritus resulting from *lichen sclerosus et atrophicus* [125]. Similarly, menthol and cooling inhibit histamine- and lichenification-evoked itch in humans [126]. Recently, Palkar et al. found that cooling and the cold mimetic menthol could inhibit both histaminergic and non-histaminergic itch pathways. Itch inhibition by cooling required the participation of TRPM8 and functional intact TRPM8-expressing afferent neurons [120]. More importantly, dry skin-associated chronic itch could also be ameliorated by cooling in mice [120]. These results suggested that TRPM8 was an itch modulator and that the TRPM8-mediated counter-stimulus activated a specific neural circuit that represented a potential cellular mechanism that could be exploited for chronic itch treatments.

### 3.7. TRPC4-Dependent Itch

Selective serotonin reuptake inhibitors (SSRIs) are among the most commonly used antidepressants prescribed and well known to elicit adverse skin reactions including rashes, urticaria, and pruritus with unknown mechanisms [127]. Recently, Lee et al. reported that HTR2B and TRPC4 were involved in SSRI-evoked pruritus [128]. Subcutaneous injections of 1 mM sertraline into mice evoked robust acute scratching, and mice treated with siRNA targeting HTR2B displayed significant reductions in sertraline-evoked itch behavior compared with mice receiving a control siRNA. Sertraline-evoked itch is also significantly attenuated by genetic ablation of TRPC4, but not TRPA1 or TRPV1 function, implicating the role of TRPC4 in SSRI-induced itch (Figure 1). It has also been reported that cutaneous administration of 100 μM sertraline induced itch through serotonin receptor 7 (HTR7) using genetic ablation and pharmacological inhibition approaches [52]. Thus, SSRI-evoked itch may arise through multiple pathways, one of which includes TRPC4. These recent findings raise the question of whether TRPC4 mediates other conditions in which pruritogenic 5-HT signaling can occur, such as AD, cholestasis, and psoriasis.

## 4. Ligands Commonly Used for Studying Itch-Related TRP Channels

TRP channel ligands are crucial tools for revealing the biological function of TRP channels in itch sensation (Table 1). Additionally, antagonists of TRPA1, TRPV1, TRPV3, TRPV4, and TRPC4 and agonists of TRPM8 can be potential drugs for treating itch and other TRP-related diseases. Therefore, the identification of potent and selective TRP channel ligands is of great importance in developing effective itch therapies.

### 4.1. TRPA1 Antagonists

HC-030031 is the most widely used TRPA1 blocker. It inhibits AITC- and formalin-evoked Ca^2+^ influx with IC_50_ values of 6.2 ± 0.2 and 5.3 ± 0.2 μM, respectively [129]. However, when tested in radioligand binding assays at 10 μM concentration, HC-030031 also displays activities against several other membrane proteins including sodium channels (40%) and sigma receptors (37%) [147]. HC030031 has been widely used to explore the TRPA1 function in itch. The application of 30 mg/kg HC030031 can alleviate scratching responses induced by the selective PAR4 agonist AYPGKF-NH_2_ (AYP) by 55% in mice [148]. Similarly, the number of scratching bouts in a mouse model of AD is significantly attenuated after the administration of HC-030031, which persists for 1 h [149]. HC030031 also inhibits scratching responses in ACD in mice, elicited by the exposure to haptens including oxazolone and urushiol, the contact allergen of poison ivy [150]. Moreover, HC030031 also suppresses scratching responses induced by the chemoattractant leukotriene B_4_ (LTB_4_) in female CD1 mice [151].

A-967079 is another commonly used TRPA1 antagonist [130]. It inhibits mouse TRPA1 with an IC_50_ value of 0.3 μM [130]. A-967079 is >1000-fold selective over other TRP channels and >150-fold selective over 75 other tested proteins [130]. A 3D structure of human TRPA1 in complex with A-967079 shows that two amino acid residues (S873 and T874) located in the fifth transmembrane domain of TRPA1 play important roles in interacting with A-967079 [152]. A-967079 effectively suppresses spontaneous scratching in mouse models of ACD chemically induced by SADBE, oxazolone, urushiol, poison ivy, and 2,4-dinitrochlorobenzene (DNCB) [30,150,153].

AP18, identified as a potent and selective TRPA1 inhibitor, suppresses Ca^2+^ responses evoked by 50 μM cinnamaldehyde with IC_50_ values of 3.1 μM and 4.5 μM for human and mouse TRPA1, respectively [131]. Importantly, at concentrations up to 50 μM, AP18 is unable to appreciably block activation of TRPV1, TRPV2, TRPV3, TRPV4, or TRPM8 [131]. Schenkel et al. identified compound 4 as a TRPA1 antagonist by using high-throughput screening [132]. Although compound 4 shows antagonist activity only at concentrations up to 25 μM, a series of modifications to compound 4 led to the discovery of compound 27 (AM-0902) [132]. Compound 27 is a potent and selective TRPA1 antagonist with an IC_50_ value of 0.024 μM for human TRPA1 and it exhibits no significant activity against TRPV1, TRPV3, TRPV4, or TRPM8 [132]. However, neither AP18 nor AM-0902 have yet been used for itch studies.

### 4.2. TRPV1 Antagonists

AMG9810 is the most widely used TRPV1 inhibitor. It is a competitive antagonist of capsaicin with an IC_50_ value of 24.5 ± 15.7 nM for human TRPV1 [133]. It blocks all known modes of TRPV1 activation, including protons, heat, and endogenous ligands [133]. AMG9810 blocks capsaicin-evoked depolarization and calcitonin gene-related peptide (CGRP) release in cultures of rat DRG neurons [133]. A recent study demonstrated that AMG9810 suppressed itch behavior elicited by subcutaneous injections of immepip dihydrobromide, a selective histamine H4 receptor agonist [70].

By using high-throughput screening of a large chemical library, Gunthorpe et al. identified a potent and selective TRPV1 antagonist, SB366791 [134]. SB366791 inhibits human TRPV1 and rat TRPV1 currents activated by 1 μM capsaicin with IC_50_ values of 5.7 ± 1.2 nM and 7.5 ± 1.8 nM, respectively [134]. SB366791 also inhibits acid- and heat-activated TRPV1 current. Importantly, 1 μM SB-366791 shows little or no activity to 47 other ion channels and G protein-coupled receptors [134]. SB366791 is a potent inhibitor of itch responses induced by PAR4 or PAR2 activation in mice [148,154].

TRPV1 antagonists have also been tested in clinical trials as anti-itch drugs. PAC-14028 is a potent TRPV1 inhibitor with an IC_50_ value of 55.0 ± 7.1 nM against capsaicin-induced calcium influx in rat DRG neurons, and it can attenuate dermatitis-associated barrier damages in *Dermatophagoides farina*- and *oxazolone*-induced mouse models of AD [135]. Recently, PAC-14028 went through multiple phase II clinical trials for determining its efficacy in relieving AD-associated pruritus (NCT02583022, NCT02052531, and NCT02565134), and an ongoing phase III trial of patients with AD is expected to be completed in late 2018 (NCT02965118). Although PAC-14028 shows a promising anti-itch effect in AD, a randomized clinical trial failed to find a symptomatic benefit of another TRPV1 antagonist, SB-705498, for histaminergic or non-histaminergic induced itch [155]. On the other hand, SB-705498 shows a promising inhibitory effect on heat-evoked pain and skin sensitization induced by capsaicin or UVB irradiation in humans [156], further suggesting distinct mechanisms might be involved in TRPV1-mediated pain and itch.

### 4.3. TRPV3 Antagonists

Hydra Biosciences is the first company to develop small molecule TRPV3 antagonists and has identified a series of TRPV3 antagonists with IC_50_ values in the range of 0.2–1 μM [157]. These antagonists show good selectivity and lack adverse effects at doses of 200 mg/kg [157]. Glenmark Pharmaceuticals is another company dedicated to developing small molecule inhibitors of TRPV3 and has described several additional TRPV3 antagonists in patent applications. For instance, GRC 15,300 is the first TRPV3 inhibitor to enter clinical phase I and clinical phase II trials for treating neuropathic pain [157,158]. However, this research is discontinued and currently there are no known ongoing clinical trials employing TRPV3 antagonists [158].

Resolvins are endogenous anti-inflammatory molecules generated by ω-3 lipid metabolism [159]. Interestingly, resolvin D1 (RvD1) and resolvin E1 (RvE1) could inhibit activities of multiple TRP channels [160]. Bang et al. demonstrated that 17R-RvD1 was a potent TRPV3 inhibitor [137]. The TRPV3-mediated Ca^2+^ influxes elicited by 4 mM camphor were blocked by 17R-RvD1 with an IC_50_ value of 398 nM [137]. More importantly, the other five TRP channels including TRPA1, TRPV1, TRPV2, TRPV4, and TRPM8 were not affected by 17R-RvD1. Thus, 17R-RvD1 represents a novel specific endogenous inhibitor of TRPV3. However, this TRPV3 antagonist has yet to be used to investigate itch.

### 4.4. TRPV4 Antagonists

Everaerts et al. identified HC-067047, a small molecule antagonist of hTRPV4 that reversibly inhibited currents mediated by human, rat, and mouse TRPV4 orthologs with IC_50_ values of 48 ± 6 nM, 133 ± 25 nM, and 17 ± 3 nM, respectively [138]. In Ca^2+^ imaging experiments, 1 μM HC-067047 completely inhibited mouse TRPV4 activated by heat, hypoosmotic solution, arachidonic acid, 4α-Phorbol 12,13-didecanoate (4α-PDD), 4α-phorbol 12,13-dihexanoate (4α-PDH), and GSK1016790A [138]. HC-067047 has a promising selectivity profile, lacking obvious effects on other ion channels except TRPM8 and hERG at submicromolar concentrations [138]. GSK2193874 is the most widely used specific antagonist of TRPV4. It inhibits human TRPV4 and rat TRPV4 with IC_50_ values of 40 nM and 2 nM, respectively [139]. Importantly, it is selective against TRPV1, TRPA1, TRPC3, TRPC6, and TRPM8 (IC_50_ > 25 μM) [139]. Both HC067047 and GSK2193874 have been used to investigate the role of TRPV4 in itch signaling. HC067047 significantly inhibits 5-HT-evoked scratching in mice [27]. Both HC067047 and GSK2193874 suppress spontaneous scratching in mouse models of acetone–diethylether–water (AEW)-induced dry skin and SADBE-induced ACD [98]. GSK205 is another small molecule TRPV4 blocker used for itch studies, although it also possesses an inhibitory effect on TRPA1 [161]. GSK205 is shown to inhibit itch elicited by localized skin warming as well as scratching responses induced by histaminergic (histamine, compound 48/80, ET-1), but not non-histaminergic (CQ) pruritogens [28].

### 4.5. TRPC4 Antagonists

Although some small molecule TRPC4 antagonists are available, most of them have off-target effects. For instance, ML204 is identified as a novel TRPC4 antagonist with an IC_50_ value of 0.96 μM [140]. However, ML204 also exhibits inhibitory effects on TRPC5 and TRPC6 channels at 10 μM [140]. Similarly, Zhu et al. identified M084 and its analogues as TRPC4 inhibitors with IC_50_ values in the range of 4.1 μM to 11.0 μM [162]. Unfortunately, all these compounds also block TRPC5 with similar IC_50_ values [162]. More recently, Just et al. reported a highly potent small molecule antagonist of TRPC4, HC-070, which inhibited hTRPC4 with an IC_50_ value of 46.0 ± 3.9 nM [141]. Although this compound is >400-fold selective over a wide range of molecular targets including ion channels, receptors, and kinases, it also inhibits hTRPC5 with an IC_50_ value of 9.3 ± 0.9 nM [141]. Selective TRPC4 blockers remain to be utilized for itch studies.

### 4.6. TRPM8 Agonists

Specific TRPM8 activators are promising candidates for treating itch since the activation of TRPM8 by cooling or chemical agonists relieves itch [125,163]. Menthol and icilin are well-known and widely used compounds activating TRPM8 with EC_50_ values of 10.4 μM and 1.4 μM, respectively [145,146]. Indeed, both menthol and icilin display excellent anti-itch effects. Menthol significantly reduces not only itch induced by mustard gas exposure but also lichen amyloidosis- and hydroxyethyl starch-evoked itch [163,164,165]. A topical icilin application relieves vulva pruritus originating from lichen sclerosus [125]. However, menthol can also activate TRPA1 and TRPV3, and icilin is also a potent TRPA1 activator [166,167].

Many other TRPM8 agonists have also been identified, including eucalyptol (1,8-cineol), WS-12, M8-Ag, CPS-369, and D-3263 [114,168,169]. Eucalyptol is the major ingredient in the essential oil of eucalyptus leaves. Eucalyptol-containing products are a widely used remedy for alleviating pain [114]. Eucalyptol is a TRPM8 agonist and can inhibit acid-induced visceral pain in a TRPM8-dependent manner [114,170]. However, eucalyptol activates TRPM8 with relatively low efficacy and potency (EC_50_ = 3.4 ± 0.4 mM) and also inhibits TRPA1 [142,143]. M8-Ag is a potent TRPM8 agonist that activates TRPM8 with an EC_50_ value of 45 nM [144]. However, M8-Ag also activates TRPA1 with an EC_50_ of >4 μM [144]. WS-12 is another well-known and widely used TRPM8 agonist with an EC_50_ of 193 nM [145,146]. WS-12 appears to be the most specific among the currently known TRPM8 agonists because it lacks actions on TRPV1, TRPV2, TRPV3, TRPV4, and TRPA1 at 1 mM [113]. The excellent potency and selectivity of WS-12 makes it a promising TRPM8 activator for treating itch.

## 5. Conclusions and Future Perspectives

Persistent itch is debilitating and severely affects quality of life. Although antihistamines, corticosteroid creams, calcineurin inhibitors, and antidepressants have been widely used to treat itch, many of them are ineffective for chronic itch and exert numerous side effects. Novel and effective anti-itch drugs are urgently needed.

Molecular and cellular mechanisms underlying the regulation of itch signaling by TRP channels have not been completely understood. Accumulating evidence now identifies TRP channels as crucial players in mediating and modulating acute and chronic itch. The role of TRP channels in itch is becoming clearer and many TRP channel ligands have been developed, yet TRP channel ligands remain to be used clinically as anti-itch drugs. Off-target effects are a risk for TRP channel ligands given their broad expression and numerous biological functions. Thus, development should proceed with caution. Nevertheless, since recent stimulating studies have provided conclusive evidence that multiple TRP channels play critical roles in the initiation and maintenance of itch signaling, the development of new and selective drugs targeting TRP channels should hold promise for the treatment of chronic itch.

## Figures and Tables

**Table 1:** Ligands of itch-related TRP channels.

Ligands	Targets	Potencies	References
HC-030031	TRPA1	IC_50_ = 6.2 and 5.3 μM for AITC- and formalin-evoked Ca^2+^ influx, respectively.	[129]
A-967079	TRPA1	IC_50_ = 0.3 μM for mTRPA1	[130]
AP18	TRPA1	IC_50_ = 3.1 μM and 4.5 μM for hTRPA1 and mTRPA1, respectively	[131]
AM-0902	TRPA1	IC_50_ = 24 nM for hTRPA1	[132]
AMG9810	TRPV1	IC_50_ = 24.5 nM for hTRPV1	[133]
SB366791	TRPV1	IC_50_ = 5.7 nM and 7.5 nM for hTRPV1 and rTRPV1, respectively	[134]
PAC-14028	TRPV1	IC_50_ = 55.0 nM for rTRPV1	[135]
SB-705498	TRPV1	IC_50_ = 3 nM for capsaicin-induced activation of hTRPV1	[136]
17R-RvD1	TRPV3	IC_50_ = 398 nM for hTRPV3	[137]
HC-067047	TRPV4	IC_50_ = 48 nM, 133 nM, and 17 nM for hTRPV4, rTRPV4, and mTRPV4, respectively	[138]
GSK2193874	TRPV4	IC_50_ = 40 nM and 2 nM for hTRPV4 and rTRPV4, respectively	[139]
ML204	TRPC4 TRPC5 TRPC6	IC_50_ = 0.96 μM for mTRPC4β and about 65% inhibition of TRPC5 and 38% inhibition of TRPC6 at 10 μM	[140]
HC-070	TRPC4 TRPC5	IC_50_ = 46.0 nM for hTRPC4 IC_50_ = 9.3 nM for hTRPC5	[141]
1,8-cineole	TRPM8 TRPA1	EC_50_ = 3.4 mM for TRPM8 IC_50_ = 3.4 mM for TRPA1	[142,143]
M8-Ag	TRPM8 TRPA1	EC_50_ = 45 nM for TRPM8 EC_50_ > 4 μM for TRPA1	[144]
WS-12	TRPM8	EC_50_ = 193 nM for hTRPM8	[145,146]

## Abbreviation

TRP	transient receptor potential
Mrgpr	Mas-related G protein-coupled receptor
TSLP	thymic stromal lymphopoietin
TSLPR	thymic stromal lymphopoietin receptor
SLIGRL	peptide Ser-Leu-Ile-Gly-Arg-Leu
BAM8-22	bovine adrenal medulla 8–22 peptide
5-HT	5-hydroxytryptamine
HTR7	5-hydroxytryptamine receptor 7
TGR5	G protein-coupled bile acid receptor Gpbar1
PLC	phospholipase C
H1R	histamine receptor 1
H2R	histamine receptor 2
H3R	histamine receptor 3
H4R	histamine receptor 4
SADBE	squaric acid dibutylester
LPA	lysophosphatidic acid
IL-31	interleukin-31
AEW	acetone–diethylether–water
CQ	chloroquine
4-HNE	4-hydroxynonenal
S1P	sphingosine 1-phosphate
S1PR3	sphingosine 1-phosphate receptor 3
OS	Olmsted syndrome
GPCR	G protein-coupled receptor
AD	atopic dermatitis
KO	knockout
PKC	protein kinase C
GRP	gastrin-releasing peptide
IL7R	interleukin-7 receptor
CHS	contact hypersensitivity
ACD	allergic contact dermatitis
LTB_4_	leukotriene B_4_
2-APB	2-aminoethoxydiphenyl borate
CIP	chronic idiopathic pruritus
ET-1	endothelin-1
SSRIs	selective serotonin reuptake inhibitors
AYP	AYPGKF-NH_2_
DNCB	dinitrochlorobenzene
CGRP	calcitonin gene-related peptide
RvD1	resolvin D1
RvE1	resolvin E1
4α-PDD	4α-Phorbol 12,13-didecanoate
4α-PDH	4α-phorbol 12,13-dihexanoate

## References

[1] Paus R., Schmelz M., Biro T., Steinhoff M. (2006). Frontiers in pruritus research: Scratching the brain for more effective itch therapy. J. Clin. Investig..

[2] Akiyama T., Carstens E. (2013). Neural processing of itch. Neuroscience.

[3] Wang H., Yosipovitch G. (2010). New insights into the pathophysiology and treatment of chronic itch in patients with end-stage renal disease, chronic liver disease, and lymphoma. Int. J. Dermatol..

[4] Boguniewicz M., Leung D.Y. (2011). Atopic dermatitis: A disease of altered skin barrier and immune dysregulation. Immunol. Rev..

[5] Bunchorntavakul C., Reddy K.R. (2012). Pruritus in chronic cholestatic liver disease. Clin. Liver Dis..

[6] Ikoma A., Steinhoff M., Stander S., Yosipovitch G., Schmelz M. (2006). The neurobiology of itch. Nat. Rev. Neurosci..

[7] Montell C., Birnbaumer L., Flockerzi V. (2002). The TRP channels, a remarkably functional family. Cell.

[8] Venkatachalam K., Montell C. (2007). TRP channels. Annu. Rev. Biochem..

[9] Ren Z., Rhyu M.R., Phan T.H., Mummalaneni S., Murthy K.S., Grider J.R., DeSimone J.A., Lyall V. (2013). TRPM5-dependent amiloride- and benzamil-insensitive NaCl chorda tympani taste nerve response. Am. J. Physiol. Gastrointest. Liver Physiol..

[10] Zheng J. (2013). Molecular mechanism of TRP channels. Compr. Physiol..

[11] Chatzigeorgiou M., Yoo S., Watson J.D., Lee W.H., Spencer W.C., Kindt K.S., Hwang S.W., Miller D.M., Treinin M., Driscoll M. (2010). Specific roles for DEG/ENaC and TRP channels in touch and thermosensation in C. elegans nociceptors. Nat. Neurosci..

[12] Wu L.J., Sweet T.B., Clapham D.E. (2010). International Union of Basic and Clinical Pharmacology. LXXVI. Current progress in the mammalian TRP ion channel family. Pharmacol. Rev..

[13] Steinhoff M., Biro T. (2009). A TR(I)P to pruritus research: Role of TRPV3 in inflammation and itch. J. Investig. Dermatol..

[14] Moore C., Gupta R., Jordt S.E., Chen Y., Liedtke W.B. (2018). Regulation of Pain and Itch by TRP Channels. Neurosci. Bull..

[15] McMahon S.B., Koltzenburg M. (1992). Itching for an explanation. Trends Neurosci..

[16] Ward L., Wright E., McMahon S.B. (1996). A comparison of the effects of noxious and innocuous counterstimuli on experimentally induced itch and pain. Pain.

[17] Liu X.Y., Liu Z.C., Sun Y.G., Ross M., Kim S., Tsai F.F., Li Q.F., Jeffry J., Kim J.Y., Loh H.H. (2011). Unidirectional cross-activation of GRPR by MOR1D uncouples itch and analgesia induced by opioids. Cell.

[18] Sun Y.G., Zhao Z.Q., Meng X.L., Yin J., Liu X.Y., Chen Z.F. (2009). Cellular basis of itch sensation. Science.

[19] Dong X., Dong X. (2018). Peripheral and Central Mechanisms of Itch. Neuron.

[20] LaMotte R.H., Dong X., Ringkamp M. (2014). Sensory neurons and circuits mediating itch. Nat. Rev. Neurosci..

[21] Han L., Ma C., Liu Q., Weng H.J., Cui Y., Tang Z., Kim Y., Nie H., Qu L., Patel K.N. (2013). A subpopulation of nociceptors specifically linked to itch. Nat. Neurosci..

[22] Luo J., Feng J., Liu S., Walters E.T., Hu H. (2015). Molecular and cellular mechanisms that initiate pain and itch. Cell. Mol. Life Sci. CMLS.

[23] Galli S.J., Nakae S., Tsai M. (2005). Mast cells in the development of adaptive immune responses. Nat. Immunol..

[24] Werfel T., Allam J.P., Biedermann T., Eyerich K., Gilles S., Guttman-Yassky E., Hoetzenecker W., Knol E., Simon H.U., Wollenberg A. (2016). Cellular and molecular immunologic mechanisms in patients with atopic dermatitis. J. Allergy Clin. Immunol..

[25] Feld M., Garcia R., Buddenkotte J., Katayama S., Lewis K., Muirhead G., Hevezi P., Plesser K., Schrumpf H., Krjutskov K. (2016). The pruritus- and TH2-associated cytokine IL-31 promotes growth of sensory nerves. J. Allergy Clin. Immunol..

[26] Cevikbas F., Wang X., Akiyama T., Kempkes C., Savinko T., Antal A., Kukova G., Buhl T., Ikoma A., Buddenkotte J. (2014). A sensory neuron-expressed IL-31 receptor mediates T helper cell-dependent itch: Involvement of TRPV1 and TRPA1. J. Allergy Clin. Immunol..

[27] Akiyama T., Ivanov M., Nagamine M., Davoodi A., Carstens M.I., Ikoma A., Cevikbas F., Kempkes C., Buddenkotte J., Steinhoff M. (2016). Involvement of TRPV4 in Serotonin-Evoked Scratching. J. Investig. Dermatol..

[28] Chen Y., Fang Q., Wang Z., Zhang J.Y., MacLeod A.S., Hall R.P., Liedtke W.B. (2016). Transient Receptor Potential Vanilloid 4 Ion Channel Functions as a Pruriceptor in Epidermal Keratinocytes to Evoke Histaminergic Itch. J. Boil. Chem..

[29] Kim S., Barry D.M., Liu X.Y., Yin S., Munanairi A., Meng Q.T., Cheng W., Mo P., Wan L., Liu S.B. (2016). Facilitation of TRPV4 by TRPV1 is required for itch transmission in some sensory neuron populations. Sci. Signal..

[30] Feng J., Yang P., Mack M.R., Dryn D., Luo J., Gong X., Liu S., Oetjen L.K., Zholos A.V., Mei Z. (2017). Sensory TRP channels contribute differentially to skin inflammation and persistent itch. Nat. Commun..

[31] Kittaka H., Tominaga M. (2017). The molecular and cellular mechanisms of itch and the involvement of TRP channels in the peripheral sensory nervous system and skin. Allergol. Int. Off. J. Jpn. Soc. Allergol..

[32] Caterina M.J., Pang Z. (2016). TRP Channels in Skin Biology and Pathophysiology. Pharmaceuticals.

[33] Sun S., Dong X. (2016). Trp channels and itch. Semin. Immunopathol..

[34] Foster S.L., Seehus C.R., Woolf C.J., Talbot S. (2017). Sense and Immunity: Context-Dependent Neuro-Immune Interplay. Front. Immunol..

[35] Zygmunt P.M., Hogestatt E.D. (2014). Trpa1. Handb. Exp. Pharmacol..

[36] Story G.M., Peier A.M., Reeve A.J., Eid S.R., Mosbacher J., Hricik T.R., Earley T.J., Hergarden A.C., Andersson D.A., Hwang S.W. (2003). ANKTM1, a TRP-like channel expressed in nociceptive neurons, is activated by cold temperatures. Cell.

[37] Jordt S.E., Bautista D.M., Chuang H.H., McKemy D.D., Zygmunt P.M., Hogestatt E.D., Meng I.D., Julius D. (2004). Mustard oils and cannabinoids excite sensory nerve fibres through the TRP channel ANKTM1. Nature.

[38] Atoyan R., Shander D., Botchkareva N.V. (2009). Non-neuronal expression of transient receptor potential type A1 (TRPA1) in human skin. J. Investig. Dermatol..

[39] Takaya J., Mio K., Shiraishi T., Kurokawa T., Otsuka S., Mori Y., Uesugi M. (2015). A Potent and Site-Selective Agonist of TRPA1. J. Am. Chem. Soc..

[40] Alpizar Y.A., Gees M., Sanchez A., Apetrei A., Voets T., Nilius B., Talavera K. (2013). Bimodal effects of cinnamaldehyde and camphor on mouse TRPA1. Pflugers Arch. Eur. J. Physiol..

[41] Macpherson L.J., Geierstanger B.H., Viswanath V., Bandell M., Eid S.R., Hwang S., Patapoutian A. (2005). The pungency of garlic: Activation of TRPA1 and TRPV1 in response to allicin. Curr. Boil..

[42] Sawada Y., Hosokawa H., Matsumura K., Kobayashi S. (2008). Activation of transient receptor potential ankyrin 1 by hydrogen peroxide. Eur. J. Neurosci..

[43] Trevisani M., Siemens J., Materazzi S., Bautista D.M., Nassini R., Campi B., Imamachi N., Andre E., Patacchini R., Cottrell G.S. (2007). 4-Hydroxynonenal, an endogenous aldehyde, causes pain and neurogenic inflammation through activation of the irritant receptor TRPA1. Proc. Natl. Acad. Sci. USA.

[44] Hu H., Bandell M., Petrus M.J., Zhu M.X., Patapoutian A. (2009). Zinc activates damage-sensing TRPA1 ion channels. Nat. Chem. Boil..

[45] Lieu T., Jayaweera G., Zhao P., Poole D.P., Jensen D., Grace M., McIntyre P., Bron R., Wilson Y.M., Krappitz M. (2014). The bile acid receptor TGR5 activates the TRPA1 channel to induce itch in mice. Gastroenterology.

[46] Liu Q., Tang Z., Surdenikova L., Kim S., Patel K.N., Kim A., Ru F., Guan Y., Weng H.J., Geng Y. (2009). Sensory neuron-specific GPCR Mrgprs are itch receptors mediating chloroquine-induced pruritus. Cell.

[47] Wilson S.R., The L., Batia L.M., Beattie K., Katibah G.E., McClain S.P., Pellegrino M., Estandian D.M., Bautista D.M. (2013). The epithelial cell-derived atopic dermatitis cytokine TSLP activates neurons to induce itch. Cell.

[48] Wilson S.R., Gerhold K.A., Bifolck-Fisher A., Liu Q., Patel K.N., Dong X., Bautista D.M. (2011). TRPA1 is required for histamine-independent, Mas-related G protein-coupled receptor-mediated itch. Nat. Neurosci..

[49] Ru F., Sun H., Jurcakova D., Herbstsomer R.A., Meixong J., Dong X., Undem B.J. (2017). Mechanisms of pruritogen-induced activation of itch nerves in isolated mouse skin. J. Physiol..

[50] Moon M.L., Joesting J.J., Lawson M.A., Chiu G.S., Blevins N.A., Kwakwa K.A., Freund G.G. (2014). The saturated fatty acid, palmitic acid, induces anxiety-like behavior in mice. Metab. Clin. Exp..

[51] Soga F., Katoh N., Inoue T., Kishimoto S. (2007). Serotonin activates human monocytes and prevents apoptosis. J. Investig. Dermatol..

[52] Morita T., McClain S.P., Batia L.M., Pellegrino M., Wilson S.R., Kienzler M.A., Lyman K., Olsen A.S., Wong J.F., Stucky C.L. (2015). HTR7 Mediates Serotonergic Acute and Chronic Itch. Neuron.

[53] Bassari R., Koea J.B. (2015). Jaundice associated pruritis: A review of pathophysiology and treatment. World J. Gastroenterol..

[54] Mela M., Mancuso A., Burroughs A.K. (2003). Review article: Pruritus in cholestatic and other liver diseases. Aliment. Pharmacol. Ther..

[55] Kirby J., Heaton K.W., Burton J.L. (1974). Pruritic effect of bile salts. Br. Med. J..

[56] Ziegler S.F., Roan F., Bell B.D., Stoklasek T.A., Kitajima M., Han H. (2013). The biology of thymic stromal lymphopoietin (TSLP). Adv. Pharmacol..

[57] Li M., Messaddeq N., Teletin M., Pasquali J.L., Metzger D., Chambon P. (2005). Retinoid X receptor ablation in adult mouse keratinocytes generates an atopic dermatitis triggered by thymic stromal lymphopoietin. Proc. Natl. Acad. Sci. USA.

[58] Ahern K., Gilmore E.S., Poligone B. (2012). Pruritus in cutaneous T-cell lymphoma: A review. J. Am. Acad. Dermatol..

[59] Han Q., Liu D., Convertino M., Wang Z., Jiang C., Kim Y.H., Luo X., Zhang X., Nackley A., Dokholyan N.V. (2018). miRNA-711 Binds and Activates TRPA1 Extracellularly to Evoke Acute and Chronic Pruritus. Neuron.

[60] Sheahan T.D., Hachisuka J., Ross S.E. (2018). Small RNAs, but Sizable Itch: TRPA1 Activation by an Extracellular MicroRNA. Neuron.

[61] Kim S.E., Patapoutian A., Grandl J. (2013). Single residues in the outer pore of TRPV1 and TRPV3 have temperature-dependent conformations. PLoS ONE.

[62] Rosenbaum T., Simon S.A., Liedtke W.B., Heller S. (2007). TRPV1 Receptors and Signal Transduction. TRP Ion Channel Function in Sensory Transduction and Cellular Signaling Cascades.

[63] Carnevale V., Rohacs T. (2016). TRPV1: A Target for Rational Drug Design. Pharmaceuticals.

[64] Brederson J.D., Kym P.R., Szallasi A. (2013). Targeting TRP channels for pain relief. Eur. J. Pharmacol..

[65] Yin S., Luo J., Qian A., Du J., Yang Q., Zhou S., Yu W., Du G., Clark R.B., Walters E.T. (2013). Retinoids activate the irritant receptor TRPV1 and produce sensory hypersensitivity. J. Clin. Investig..

[66] Shim W.S., Oh U. (2008). Histamine-induced itch and its relationship with pain. Mol. Pain.

[67] Magerl W., Westerman R.A., Mohner B., Handwerker H.O. (1990). Properties of transdermal histamine iontophoresis: Differential effects of season, gender, and body region. J. Investig. Dermatol..

[68] Ohsawa Y., Hirasawa N. (2014). The role of histamine H1 and H4 receptors in atopic dermatitis: From basic research to clinical study. Allergol. Int. Off. J. Jpn. Soc. Allergol..

[69] Shim W.S., Tak M.H., Lee M.H., Kim M., Kim M., Koo J.Y., Lee C.H., Kim M., Oh U. (2007). TRPV1 mediates histamine-induced itching via the activation of phospholipase A2 and 12-lipoxygenase. J. Neurosci. Off. J. Soc. Neurosci..

[70] Jian T., Yang N., Yang Y., Zhu C., Yuan X., Yu G., Wang C., Wang Z., Shi H., Tang M. (2016). TRPV1 and PLC Participate in Histamine H4 Receptor-Induced Itch. Neural Plast..

[71] Morales-Lazaro S.L., Llorente I., Sierra-Ramirez F., Lopez-Romero A.E., Ortiz-Renteria M., Serrano-Flores B., Simon S.A., Islas L.D., Rosenbaum T. (2016). Inhibition of TRPV1 channels by a naturally occurring omega-9 fatty acid reduces pain and itch. Nat. Commun..

[72] Kremer A.E., Martens J.J., Kulik W., Rueff F., Kuiper E.M., van Buuren H.R., van Erpecum K.J., Kondrackiene J., Prieto J., Rust C. (2010). Lysophosphatidic acid is a potential mediator of cholestatic pruritus. Gastroenterology.

[73] Hashimoto T., Ohata H., Momose K. (2004). Itch-scratch responses induced by lysophosphatidic acid in mice. Pharmacology.

[74] Kittaka H., Uchida K., Fukuta N., Tominaga M. (2017). Lysophosphatidic acid-induced itch is mediated by signalling of LPA5 receptor, phospholipase D and TRPA1/TRPV1. J. Physiol..

[75] Nieto-Posadas A., Picazo-Juarez G., Llorente I., Jara-Oseguera A., Morales-Lazaro S., Escalante-Alcalde D., Islas L.D., Rosenbaum T. (2011). Lysophosphatidic acid directly activates TRPV1 through a C-terminal binding site. Nat. Chem. Boil..

[76] Mastrolonardo M., Diaferio A. (2002). Topical immunotherapy with squaric acid dibutylester: Unusual hair pigmentary changes in two cases of alopecia areata. J. Eur. Acad. Dermatol. Venereol. JEADV.

[77] Zoller M., Freyschmidt-Paul P., Vitacolonna M., McElwee K.J., Hummel S., Hoffmann R. (2004). Chronic delayed-type hypersensitivity reaction as a means to treat alopecia areata. Clin. Exp. Immunol..

[78] Qu L., Fan N., Ma C., Wang T., Han L., Fu K., Wang Y., Shimada S.G., Dong X., LaMotte R.H. (2014). Enhanced excitability of MRGPRA3- and MRGPRD-positive nociceptors in a model of inflammatory itch and pain. Brain.

[79] Yosipovitch G., Bernhard J.D. (2013). Clinical practice. Chronic pruritus. N. Engl. J. Med..

[80] Suto H., Matsuda H., Mitsuishi K., Hira K., Uchida T., Unno T., Ogawa H., Ra C. (1999). NC/Nga mice: A mouse model for atopic dermatitis. Int. Arch. Allergy Immunol..

[81] Cevikbas F., Kempkes C., Buhl T., Mess C., Buddenkotte J., Steinhoff M., Carstens E., Akiyama T. (2014). Role of Interleukin-31 and Oncostatin M in Itch and Neuroimmune Communication. Itch: Mechanisms and Treatment.

[82] Hill R.Z., Hoffman B.U., Morita T., Campos S.M., Lumpkin E.A., Brem R.B., Bautista D.M. (2018). The signaling lipid sphingosine 1-phosphate regulates mechanical pain. eLife.

[83] Weth D., Benetti C., Rauch C., Gstraunthaler G., Schmidt H., Geisslinger G., Sabbadini R., Proia R.L., Kress M. (2015). Activated platelets release sphingosine 1-phosphate and induce hypersensitivity to noxious heat stimuli in vivo. Front. Neurosci..

[84] Janes K., Little J.W., Li C., Bryant L., Chen C., Chen Z., Kamocki K., Doyle T., Snider A., Esposito E. (2014). The development and maintenance of paclitaxel-induced neuropathic pain require activation of the sphingosine 1-phosphate receptor subtype 1. J. Boil. Chem..

[85] Hill R.Z., Morita T., Brem R.B., Bautista D.M. (2018). S1PR3 mediates itch and pain via distinct TRP channel-dependent pathways. J. Neurosci. Off. J. Soc. Neurosci..

[86] Xu H., Ramsey I.S., Kotecha S.A., Moran M.M., Chong J.A., Lawson D., Ge P., Lilly J., Silos-Santiago I., Xie Y. (2002). TRPV3 is a calcium-permeable temperature-sensitive cation channel. Nature.

[87] Bang S., Yoo S., Yang T.J., Cho H., Hwang S.W. (2010). Farnesyl pyrophosphate is a novel pain-producing molecule via specific activation of TRPV3. J. Boil. Chem..

[88] Hu H., Grandl J., Bandell M., Petrus M., Patapoutian A. (2009). Two amino acid residues determine 2-APB sensitivity of the ion channels TRPV3 and TRPV4. Proc. Natl. Acad. Sci. USA.

[89] Feketa V.V., Marrelli S.P. (2015). Systemic Administration of the TRPV3 Ion Channel Agonist Carvacrol Induces Hypothermia in Conscious Rodents. PLoS ONE.

[90] Vogt-Eisele A.K., Weber K., Sherkheli M.A., Vielhaber G., Panten J., Gisselmann G., Hatt H. (2007). Monoterpenoid agonists of TRPV3. Br. J. Pharmacol..

[91] Ortar G., Morera L., Moriello A.S., Morera E., Nalli M., Di Marzo V., De Petrocellis L. (2012). Modulation of thermo-transient receptor potential (thermo-TRP) channels by thymol-based compounds. Bioorg. Med. Chem. Lett..

[92] Stotz S.C., Vriens J., Martyn D., Clardy J., Clapham D.E. (2008). Citral sensing by Transient [corrected] receptor potential channels in dorsal root ganglion neurons. PLoS ONE.

[93] Peier A.M., Reeve A.J., Andersson D.A., Moqrich A., Earley T.J., Hergarden A.C., Story G.M., Colley S., Hogenesch J.B., McIntyre P. (2002). A heat-sensitive TRP channel expressed in keratinocytes. Science.

[94] Moqrich A., Hwang S.W., Earley T.J., Petrus M.J., Murray A.N., Spencer K.S., Andahazy M., Story G.M., Patapoutian A. (2005). Impaired thermosensation in mice lacking TRPV3, a heat and camphor sensor in the skin. Science.

[95] Xu H., Delling M., Jun J.C., Clapham D.E. (2006). Oregano, thyme and clove-derived flavors and skin sensitizers activate specific TRP channels. Nat. Neurosci..

[96] Yamamoto-Kasai E., Yasui K., Shichijo M., Sakata T., Yoshioka T. (2013). Impact of TRPV3 on the development of allergic dermatitis as a dendritic cell modulator. Exp. Dermatol..

[97] Yang Y.S., Cho S.I., Choi M.G., Choi Y.H., Kwak I.S., Park C.W., Kim H.O. (2015). Increased expression of three types of transient receptor potential channels (TRPA1, TRPV4 and TRPV3) in burn scars with post-burn pruritus. Acta Dermato-Venereol..

[98] Luo J., Feng J., Yu G., Yang P., Mack M.R., Du J., Yu W., Qian A., Zhang Y., Liu S. (2018). Transient receptor potential vanilloid 4-expressing macrophages and keratinocytes contribute differentially to allergic and nonallergic chronic itch. J. Allergy Clin. Immunol..

[99] Cheng X., Jin J., Hu L., Shen D., Dong X.P., Samie M.A., Knoff J., Eisinger B., Liu M.L., Huang S.M. (2010). TRP channel regulates EGFR signaling in hair morphogenesis and skin barrier formation. Cell.

[100] Mickle A.D., Shepherd A.J., Mohapatra D.P. (2015). Sensory TRP channels: The key transducers of nociception and pain. Prog. Mol. Boil. Transl. Sci..

[101] Nilius B., Biro T., Owsianik G. (2014). TRPV3: Time to decipher a poorly understood family member!. J. Physiol..

[102] Asakawa M., Yoshioka T., Matsutani T., Hikita I., Suzuki M., Oshima I., Tsukahara K., Arimura A., Horikawa T., Hirasawa T. (2006). Association of a mutation in TRPV3 with defective hair growth in rodents. J. Investig. Dermatol..

[103] Xiao R., Tian J., Tang J., Zhu M.X. (2008). The TRPV3 mutation associated with the hairless phenotype in rodents is constitutively active. Cell Calcium.

[104] Nagai H., Takaoka Y., Sugano A., Nakamachi Y., Kawano S., Nishigori C. (2017). Identification of a heterozygous p.Gly568Val missense mutation in the TRPV3 gene in a Japanese patient with Olmsted syndrome: In silico analysis of TRPV3. J. Dermatol..

[105] Atherton D.J., Sutton C., Jones B.M. (1990). Mutilating palmoplantar keratoderma with periorificial keratotic plaques (Olmsted’s syndrome). Br. J. Dermatol..

[106] Choi J.Y., Kim S.E., Lee S.E., Kim S.C. (2018). Olmsted Syndrome Caused by a Heterozygous p.Gly568Val Missense Mutation in TRPV3 Gene. Yonsei Med. J..

[107] Shibasaki K., Sugio S., Takao K., Yamanaka A., Miyakawa T., Tominaga M., Ishizaki Y. (2015). TRPV4 activation at the physiological temperature is a critical determinant of neuronal excitability and behavior. Pflugers Arch. Eur. J. Physiol..

[108] Sokabe T., Tominaga M. (2010). The TRPV4 cation channel: A molecule linking skin temperature and barrier function. Commun. Integr. Boil..

[109] Mamenko M., Zaika O., Boukelmoune N., O’Neil R.G., Pochynyuk O. (2015). Deciphering physiological role of the mechanosensitive TRPV4 channel in the distal nephron. Am. J. Physiol. Ren. Physiol..

[110] Moore C., Cevikbas F., Pasolli H.A., Chen Y., Kong W., Kempkes C., Parekh P., Lee S.H., Kontchou N.A., Yeh I. (2013). UVB radiation generates sunburn pain and affects skin by activating epidermal TRPV4 ion channels and triggering endothelin-1 signaling. Proc. Natl. Acad. Sci. USA.

[111] Clapham D.E., Julius D., Montell C., Schultz G. (2005). International Union of Pharmacology. XLIX. Nomenclature and structure-function relationships of transient receptor potential channels. Pharmacol. Rev..

[112] Raddatz N., Castillo J.P., Gonzalez C., Alvarez O., Latorre R. (2014). Temperature and voltage coupling to channel opening in transient receptor potential melastatin 8 (TRPM8). J. Boil. Chem..

[113] Sherkheli M.A., Gisselmann G., Vogt-Eisele A.K., Doerner J.F., Hatt H. (2008). Menthol derivative WS-12 selectively activates transient receptor potential melastatin-8 (TRPM8) ion channels. Pak. J. Pharm. Sci..

[114] Caceres A.I., Liu B., Jabba S.V., Achanta S., Morris J.B., Jordt S.E. (2017). Transient Receptor Potential Cation Channel Subfamily M Member 8 channels mediate the anti-inflammatory effects of eucalyptol. Br. J. Pharmacol..

[115] Bautista D.M., Siemens J., Glazer J.M., Tsuruda P.R., Basbaum A.I., Stucky C.L., Jordt S.E., Julius D. (2007). The menthol receptor TRPM8 is the principal detector of environmental cold. Nature.

[116] Babes A., Ciobanu A.C., Neacsu C., Babes R.M. (2011). TRPM8, a sensor for mild cooling in mammalian sensory nerve endings. Curr. Pharm. Biotechnol..

[117] Denda M., Tsutsumi M., Denda S. (2010). Topical application of TRPM8 agonists accelerates skin permeability barrier recovery and reduces epidermal proliferation induced by barrier insult: Role of cold-sensitive TRP receptors in epidermal permeability barrier homoeostasis. Exp. Dermatol..

[118] Lagerstrom M.C., Rogoz K., Abrahamsen B., Persson E., Reinius B., Nordenankar K., Olund C., Smith C., Mendez J.A., Chen Z.F. (2010). VGLUT2-dependent sensory neurons in the TRPV1 population regulate pain and itch. Neuron.

[119] Liu Y., Abdel Samad O., Zhang L., Duan B., Tong Q., Lopes C., Ji R.R., Lowell B.B., Ma Q. (2010). VGLUT2-dependent glutamate release from nociceptors is required to sense pain and suppress itch. Neuron.

[120] Palkar R., Ongun S., Catich E., Li N., Borad N., Sarkisian A., McKemy D.D. (2018). Cooling Relief of Acute and Chronic Itch Requires TRPM8 Channels and Neurons. J. Investig. Dermatol..

[121] Liu B., Jordt S.E. (2018). Cooling the Itch via TRPM8. J. Investig. Dermatol..

[122] Proudfoot C.J., Garry E.M., Cottrell D.F., Rosie R., Anderson H., Robertson D.C., Fleetwood-Walker S.M., Mitchell R. (2006). Analgesia mediated by the TRPM8 cold receptor in chronic neuropathic pain. Curr. Boil..

[123] Stander S., Augustin M., Roggenkamp D., Blome C., Heitkemper T., Worthmann A.C., Neufang G. (2017). Novel TRPM8 agonist cooling compound against chronic itch: Results from a randomized, double-blind, controlled, pilot study in dry skin. J. Eur. Acad. Dermatol. Venereol. JEADV.

[124] Naono-Nakayama R., Sunakawa N., Ikeda T., Nishimori T. (2010). Differential effects of substance P or hemokinin-1 on transient receptor potential channels, TRPV1, TRPA1 and TRPM8, in the rat. Neuropeptides.

[125] Han J.H., Choi H.K., Kim S.J. (2012). Topical TRPM8 agonist (icilin) relieved vulva pruritus originating from lichen sclerosus et atrophicus. Acta Dermato-Venereol..

[126] Bromm B., Scharein E., Darsow U., Ring J. (1995). Effects of menthol and cold on histamine-induced itch and skin reactions in man. Neurosci. Lett..

[127] Warnock J.K., Morris D.W. (2002). Adverse cutaneous reactions to antidepressants. Am. J. Clin. Dermatol..

[128] Lee S.H., Cho P.S., Tonello R., Lee H.K., Jang J.H., Park G.Y., Hwang S.W., Park C.K., Jung S.J., Berta T. (2018). Peripheral serotonin receptor 2B and transient receptor potential channel 4 mediate pruritus to serotonergic antidepressants in mice. J. Allergy Clin. Immunol..

[129] McNamara C.R., Mandel-Brehm J., Bautista D.M., Siemens J., Deranian K.L., Zhao M., Hayward N.J., Chong J.A., Julius D., Moran M.M. (2007). TRPA1 mediates formalin-induced pain. Proc. Natl. Acad. Sci. USA.

[130] Chen J., Joshi S.K., DiDomenico S., Perner R.J., Mikusa J.P., Gauvin D.M., Segreti J.A., Han P., Zhang X.F., Niforatos W. (2011). Selective blockade of TRPA1 channel attenuates pathological pain without altering noxious cold sensation or body temperature regulation. Pain.

[131] Petrus M., Peier A.M., Bandell M., Hwang S.W., Huynh T., Olney N., Jegla T., Patapoutian A. (2007). A role of TRPA1 in mechanical hyperalgesia is revealed by pharmacological inhibition. Mol. Pain.

[132] Schenkel L.B., Olivieri P.R., Boezio A.A., Deak H.L., Emkey R., Graceffa R.F., Gunaydin H., Guzman-Perez A., Lee J.H., Teffera Y. (2016). Optimization of a Novel Quinazolinone-Based Series of Transient Receptor Potential A1 (TRPA1) Antagonists Demonstrating Potent in Vivo Activity. J. Med. Chem..

[133] Gavva N.R., Tamir R., Qu Y., Klionsky L., Zhang T.J., Immke D., Wang J., Zhu D., Vanderah T.W., Porreca F. (2005). AMG 9810 [(E)-3-(4-t-butylphenyl)-N-(2,3-dihydrobenzo[b][1,4] dioxin-6-yl)acrylamide], a novel vanilloid receptor 1 (TRPV1) antagonist with antihyperalgesic properties. J. Pharmacol. Exp. Ther..

[134] Gunthorpe M.J., Rami H.K., Jerman J.C., Smart D., Gill C.H., Soffin E.M., Luis Hannan S., Lappin S.C., Egerton J., Smith G.D. (2004). Identification and characterisation of SB-366791, a potent and selective vanilloid receptor (VR1/TRPV1) antagonist. Neuropharmacology.

[135] Yun J.W., Seo J.A., Jeong Y.S., Bae I.H., Jang W.H., Lee J., Kim S.Y., Shin S.S., Woo B.Y., Lee K.W. (2011). TRPV1 antagonist can suppress the atopic dermatitis-like symptoms by accelerating skin barrier recovery. J. Dermatol. Sci..

[136] Gunthorpe M.J., Hannan S.L., Smart D., Jerman J.C., Arpino S., Smith G.D., Brough S., Wright J., Egerton J., Lappin S.C. (2007). Characterization of SB-705498, a potent and selective vanilloid receptor-1 (VR1/TRPV1) antagonist that inhibits the capsaicin-, acid-, and heat-mediated activation of the receptor. J. Pharmacol. Exp. Ther..

[137] Bang S., Yoo S., Yang T.J., Cho H., Hwang S.W. (2012). 17(R)-resolvin D1 specifically inhibits transient receptor potential ion channel vanilloid 3 leading to peripheral antinociception. Br. J. Pharmacol..

[138] Everaerts W., Zhen X., Ghosh D., Vriens J., Gevaert T., Gilbert J.P., Hayward N.J., McNamara C.R., Xue F., Moran M.M. (2010). Inhibition of the cation channel TRPV4 improves bladder function in mice and rats with cyclophosphamide-induced cystitis. Proc. Natl. Acad. Sci. USA.

[139] Cheung M., Bao W., Behm D.J., Brooks C.A., Bury M.J., Dowdell S.E., Eidam H.S., Fox R.M., Goodman K.B., Holt D.A. (2017). Discovery of GSK2193874: An Orally Active, Potent, and Selective Blocker of Transient Receptor Potential Vanilloid 4. ACS Med. Chem. Lett..

[140] Miller M., Shi J., Zhu Y., Kustov M., Tian J.B., Stevens A., Wu M., Xu J., Long S., Yang P. (2011). Identification of ML204, a novel potent antagonist that selectively modulates native TRPC4/C5 ion channels. J. Boil. Chem..

[141] Just S., Chenard B.L., Ceci A., Strassmaier T., Chong J.A., Blair N.T., Gallaschun R.J., Del Camino D., Cantin S., D’Amours M. (2018). Treatment with HC-070, a potent inhibitor of TRPC4 and TRPC5, leads to anxiolytic and antidepressant effects in mice. PLoS ONE.

[142] McKemy D.D., Neuhausser W.M., Julius D. (2002). Identification of a cold receptor reveals a general role for TRP channels in thermosensation. Nature.

[143] Takaishi M., Fujita F., Uchida K., Yamamoto S., Sawada Shimizu M., Hatai Uotsu C., Shimizu M., Tominaga M. (2012). 1,8-cineole, a TRPM8 agonist, is a novel natural antagonist of human TRPA1. Mol. Pain.

[144] Patel R., Goncalves L., Leveridge M., Mack S.R., Hendrick A., Brice N.L., Dickenson A.H. (2014). Anti-hyperalgesic effects of a novel TRPM8 agonist in neuropathic rats: A comparison with topical menthol. Pain.

[145] Bodding M., Wissenbach U., Flockerzi V. (2007). Characterisation of TRPM8 as a pharmacophore receptor. Cell Calcium.

[146] Noyer L., Grolez G.P., Prevarskaya N., Gkika D., Lemonnier L. (2018). TRPM8 and prostate: A cold case?. Pflugers Arch. Eur. J. Physiol..

[147] Chen J., Hackos D.H. (2015). TRPA1 as a drug target—Promise and challenges. Naunyn-Schmiedeberg’s Arch. Pharmacol..

[148] Patricio E.S., Costa R., Figueiredo C.P., Gers-Barlag K., Bicca M.A., Manjavachi M.N., Segat G.C., Gentry C., Luiz A.P., Fernandes E.S. (2015). Mechanisms Underlying the Scratching Behavior Induced by the Activation of Proteinase-Activated Receptor-4 in Mice. J. Investig. Dermatol..

[149] Oh M.H., Oh S.Y., Lu J., Lou H., Myers A.C., Zhu Z., Zheng T. (2013). TRPA1-dependent pruritus in IL-13-induced chronic atopic dermatitis. J. Immunol..

[150] Liu B., Escalera J., Balakrishna S., Fan L., Caceres A.I., Robinson E., Sui A., McKay M.C., McAlexander M.A., Herrick C.A. (2013). TRPA1 controls inflammation and pruritogen responses in allergic contact dermatitis. FASEB J. Off. Publ. Fed. Am. Soc. Exp. Boil..

[151] Fernandes E.S., Vong C.T., Quek S., Cheong J., Awal S., Gentry C., Aubdool A.A., Liang L., Bodkin J.V., Bevan S. (2013). Superoxide generation and leukocyte accumulation: Key elements in the mediation of leukotriene B(4)-induced itch by transient receptor potential ankyrin 1 and transient receptor potential vanilloid 1. FASEB J. Off. Publ. Fed. Am. Soc. Exp. Boil..

[152] Saito S., Tominaga M. (2017). Evolutionary tuning of TRPA1 and TRPV1 thermal and chemical sensitivity in vertebrates. Temperature.

[153] Saarnilehto M., Chapman H., Savinko T., Lindstedt K., Lauerma A.I., Koivisto A. (2014). Contact sensitizer 2,4-dinitrochlorobenzene is a highly potent human TRPA1 agonist. Allergy.

[154] Costa R., Marotta D.M., Manjavachi M.N., Fernandes E.S., Lima-Garcia J.F., Paszcuk A.F., Quintao N.L., Juliano L., Brain S.D., Calixto J.B. (2008). Evidence for the role of neurogenic inflammation components in trypsin-elicited scratching behaviour in mice. Br. J. Pharmacol..

[155] Gibson R.A., Robertson J., Mistry H., McCallum S., Fernando D., Wyres M., Yosipovitch G. (2014). A randomised trial evaluating the effects of the TRPV1 antagonist SB705498 on pruritus induced by histamine, and cowhage challenge in healthy volunteers. PLoS ONE.

[156] Chizh B.A., O’Donnell M.B., Napolitano A., Wang J., Brooke A.C., Aylott M.C., Bullman J.N., Gray E.J., Lai R.Y., Williams P.M. (2007). The effects of the TRPV1 antagonist SB-705498 on TRPV1 receptor-mediated activity and inflammatory hyperalgesia in humans. Pain.

[157] Reilly R.M., Kym P.R. (2011). Analgesic potential of TRPV3 antagonists. Curr. Top. Med. Chem..

[158] Broad L.M., Mogg A.J., Eberle E., Tolley M., Li D.L., Knopp K.L. (2016). TRPV3 in Drug Development. Pharmaceuticals.

[159] Ariel A., Serhan C.N. (2007). Resolvins and protectins in the termination program of acute inflammation. Trends Immunol..

[160] Xu Z.Z., Zhang L., Liu T., Park J.Y., Berta T., Yang R., Serhan C.N., Ji R.R. (2010). Resolvins RvE1 and RvD1 attenuate inflammatory pain via central and peripheral actions. Nat. Med..

[161] Kanju P., Chen Y., Lee W., Yeo M., Lee S.H., Romac J., Shahid R., Fan P., Gooden D.M., Simon S.A. (2016). Small molecule dual-inhibitors of TRPV4 and TRPA1 for attenuation of inflammation and pain. Sci. Rep..

[162] Zhu Y., Lu Y., Qu C., Miller M., Tian J., Thakur D.P., Zhu J., Deng Z., Hu X., Wu M. (2015). Identification and optimization of 2-aminobenzimidazole derivatives as novel inhibitors of TRPC4 and TRPC5 channels. Br. J. Pharmacol..

[163] Panahi Y., Davoodi S.M., Khalili H., Dashti-Khavidaki S., Bigdeli M. (2007). Phenol and menthol in the treatment of chronic skin lesions following mustard gas exposure. Singap. Med. J..

[164] Frolich M., Enk A., Diepgen T.L., Weisshaar E. (2009). Successful treatment of therapy-resistant pruritus in lichen amyloidosis with menthol. Acta Dermato-Venereol..

[165] Haught J.M., Jukic D.M., English J.C. (2008). Hydroxyethyl starch-induced pruritus relieved by a combination of menthol and camphor. J. Am. Acad. Dermatol..

[166] Macpherson L.J., Hwang S.W., Miyamoto T., Dubin A.E., Patapoutian A., Story G.M. (2006). More than cool: Promiscuous relationships of menthol and other sensory compounds. Mol. Cell. Neurosci..

[167] Rawls S.M., Gomez T., Ding Z., Raffa R.B. (2007). Differential behavioral effect of the TRPM8/TRPA1 channel agonist icilin (AG-3-5). Eur. J. Pharmacol..

[168] Gardiner J.C., Kirkup A.J., Curry J., Humphreys S., O’Regan P., Postlethwaite M., Young K.C., Kitching L., Ethell B.T., Winpenny D. (2014). The role of TRPM8 in the Guinea-pig bladder-cooling reflex investigated using a novel TRPM8 antagonist. Eur. J. Pharmacol..

[169] DeFalco J., Duncton M.A., Emerling D. (2011). TRPM8 biology and medicinal chemistry. Curr. Top. Med. Chem..

[170] Liu B., Fan L., Balakrishna S., Sui A., Morris J.B., Jordt S.E. (2013). TRPM8 is the principal mediator of menthol-induced analgesia of acute and inflammatory pain. Pain.

